# Neural correlates of creative insight: Amplitude of low-frequency fluctuation of resting-state brain activity predicts creative insight

**DOI:** 10.1371/journal.pone.0203071

**Published:** 2018-08-30

**Authors:** Jiabao Lin, Xuan Cui, Xiaoying Dai, Yajue Chen, Lei Mo

**Affiliations:** 1 Center for Studies of Psychological Application, Guangdong Key Laboratory of Mental Health and Cognitive Science, School of Psychology, South China Normal University, Guangzhou, China; 2 Guangdong Provincial Key Laboratory of Mental Health and Cognitive Science, South China Normal University, Guangzhou, China; Banner Alzheimer's Institute, UNITED STATES

## Abstract

Creative insight has attracted much attention across cultures. Although previous studies have explored the neural correlates of creative insight by functional magnetic resonance imaging (fMRI), little is known about intrinsic resting-state brain activity associated with creative insight. In the present study, we used amplitude of low-frequency fluctuation (ALFF) as an index in resting-state fMRI (rs-fMRI) to identify brain regions involved in individual differences in creative insight, which was measured by the response time of creative Chinese character chunk decomposition. Our results showed that ALFF in the superior frontal gyrus (SFG) positively predicted creative insight, while ALFF in the middle cingulate cortex/insula cortex (MCC/IC), superior temporal gyrus/angular gyrus (STG/AG), anterior cingulate cortex/caudate nucleus (ACC/CN), and culmen/declive (CU/DC) negatively predicted creative insight. Moreover, these findings indicate that spontaneous brain activity in multiple regions related to breaking mental sets, solutions exploring, evaluation of novel solutions, forming task-related associations, and emotion experience contributes to creative insight. In conclusion, the present study provides new evidence to further understand the cognitive processing and neural correlates of creative insight.

## Introduction

Creative insight is a sudden comprehension that can lead to a new interpretation of a situation and yield the solution to a problem [1–4]. The importance of creative insight has long been demonstrated and creative insight contributes a lot to problem solving, artistic performance, and scientific innovation [5–7]. Therefore, creative insight is a hot topic of intense study in multiple fields. Previous behavioral studies have investigated the characteristics of creative insight, including influencing factors [6, 8], phases [9], and cognitive mechanism [10]. However, the precise neural correlates of creative insight remain poorly understood.

Recently, several task-based functional magnetic resonance imaging (fMRI) studies have explored the neural correlates of creative insight. For instance, in an fMRI study, Jung-Beeman et al. [7] used a type of problem called compound remote associates (CRA) to investigate the neural correlates of creative insight. The CRA problem consists of three words (e.g., pine, crab, sauce), and participants were instructed to think of a single word (e.g., apple) that can form compound phrases (e.g., pineapple, crabapple, and applesauce) with each of the three problem words. This problem can be solved by insight or analytic thinking. The author found that insight solutions were associated with brain activity in superior temporal gyrus (STG). Using a similar remote association task, Subramaniam et al. [11] detected that anterior cingulate cortex (ACC) showed sensitivity to creative insight. In another type of insight problem solving (e.g., solving Chinese riddles), Qiu et al. [12] found that the inferior frontal gyrus (IFG), middle frontal gyrus (MFG) and precuneus were involved in the creative insight. And Zhao et al. [13] detected that the insight condition activated the regions of ACC and MFG. Moreover, with chunk decomposition task, a specific task to study the creative insight, several studies showed that superior frontal gyrus (SFG), IFG, and inferior parietal lobule had greater activations in the creative insight condition [14–16]. These findings indicated that the lateral prefrontal cortex, STG, and ACC may be important for creative insight in a particular task. However, task-related fMRI studies have an obvious drawback that a particular task only activates particular regions. Thus, it was necessarily to elucidate the intrinsic brain mechanisms of creative insight, which tends not to be task-specific.

Previous studies indicated that systematic and stable intrinsic brain activity, especially for the resting-state brain activity, were associated with aspects of personality, intelligence, cognition processes, and neurological disorder [17–22]. For example, Kumari et al. [17] examined the neural correlates of personality dimension of introversion-extraversion, assessed with the Eysenck Personality Questionnaire, and found that introversion-extraversion scores were negatively associated with resting-state fMRI (rs-fMRI) signals in the thalamus. An EEG study showed that general intelligence was correlated with faster processing times in frontal cortex [18]. Another rs-fMRI study indicated that brain regions within the default mode network typically showed deactivations during goal-directed tasks [21]. Moreover, altered brain activity in children with attention deficit hyperactivity disorder was revealed by rs-fMRI [19]. Thus, these findings suggest the possibility that resting-state neural activity may also be correlated with insightful problem solving. However, few studies have focused on the linkage between the insightful problem-solving and intrinsic brain activity.

Amplitude of low-frequency fluctuation (ALFF) of the blood oxygenation level-dependent (BOLD) signal in the rs-fMRI was considered to be physiologically meaningful and related to spontaneous neural activity [23], and the ALFF measures the total power of a given time course within a specific frequency range (e.g., 0.01–0.08 Hz), with high test-retest reliability [24]. Previous studies had defined ALFF as an effective index to reflect the neural activity in the resting state [23, 25, 26]. For example, Huang et al. [23] found that schizophrenia patients exhibited significantly higher ALFF in the bilateral putamen compared to the healthy controls. Yin et al. [25] detected that patients with posttraumatic stress disorder showed decreased ALFF values in right lingual gyrus and insula than the healthy control. Han et al. [26] found that compared to controls, the amnestic mild cognitive impairment patients had decreased ALFF values in the posterior cingulate cortex and hippocampus. Therefore, a standard measure of ALFF was used to explore the neural basis of creative insight in this research.

Chunk decomposition refers to decomposing familiar patterns into basic elements so that they can be reorganized in a novel and meaningful manner, suggesting the possibility for solving problems which seem impossible [14]. Previous studies have confirmed that chunk decomposition is a specific form of insightful problem-solving and reflects feature of human creativity [8, 14, 15, 27–32]. The matchstick arithmetic problem was first used to investigate chunk decomposition [33]. Participants were asked to move only one stick to transform the fault arithmetic statement into a true equation. Results showed that participants were easy to transform the equation ‘IV = III+III’ to ‘VI = III+III’, which was defined as non-insightful problem-solving. However, participants were difficult to transform the equation ‘XI = III+III’ to ‘VI = III + III’, which was defined as insightful problem-solving. Since the matchstick arithmetic problem cannot provide a sufficient variety of trials for neuroimaging study, researchers developed a new chunk decomposition task using Chinese characters as the materials, which required people to transform a Chinese character into another by removing some parts [14, 34]. Similarly, the radical-level decomposition was easy for participants and defined as non-insightful problem-solving. While the stroke-level chunk decomposition was novel for participants and defined as insightful problem-solving. Details of the Chinese character chunk decomposition task have been reported elsewhere [14, 31, 34].

In this study, we used a revised Chinese character chunk decomposition task as the insight problem-solving task to explore the neural mechanisms of creative insight. Considering the experimental material difficulty may potentially influence the performance of participants, we designed two types of creative Chinese character chunk decomposition varying in difficulty: creative chunk decomposition-low level (CCDL) and creative chunk decomposition-high level (CCDH). The CCDL was defined as requiring participants to transform a Chinese character into a new one by removing a particular part, such as a stroke, which was novel to participants but could be solved with a high probability of success. While in the CCDH condition, participants were asked to transform a character into another one by removing an intact character, like an isolated character, which was much more novel to participants and could be solved with a low probability of success. Previous studies have confirmed that chunk decomposition was a specific form of insightful problem-solving through behavioral data such as response time (RT) and RT could partly reflect the degree of creative insight [14, 33]. For each participant, to reduce the impact of material difficulty, we first calculated the mean RT by averaging the RT of the CCDL and CCDH. Then we used this mean RT to represent the creative insight score of each participant. Longer (shorter) RT indicated lower (higher) creative insight score. Finally, we computed the correlation between creative insight and ALFF across the whole brain to see which brain regions were linked to creative insight.

Previous task-fMRI studies suggested that the prefrontal cortex, STG and ACC may be important for creative insight [7, 11, 13–16]. Based on these findings, we hypothesized that individual differences in creative insight would be significantly correlated with the ALFF in some regions of the prefrontal cortex (e.g., SFG), STG, and ACC which have been linked to conflict detection and monitoring, spatially oriented processing, object-related exploration, reasoning, and affective function.

## Methods

### Participants

We recruited 62 right-handed and paid volunteers (28 M/34 F, aged 18–27 years old, 21.02 ± 2.08 years old) in this study. Participants were healthy, had normal or corrected-to-normal vision, and reported no history of neurological or psychiatric disorders. The study protocol was approved by the Research Ethics Review Board of South China Normal University. Written informed consent was obtained from each participant prior to the study.

### Materials

**Fig 1:**
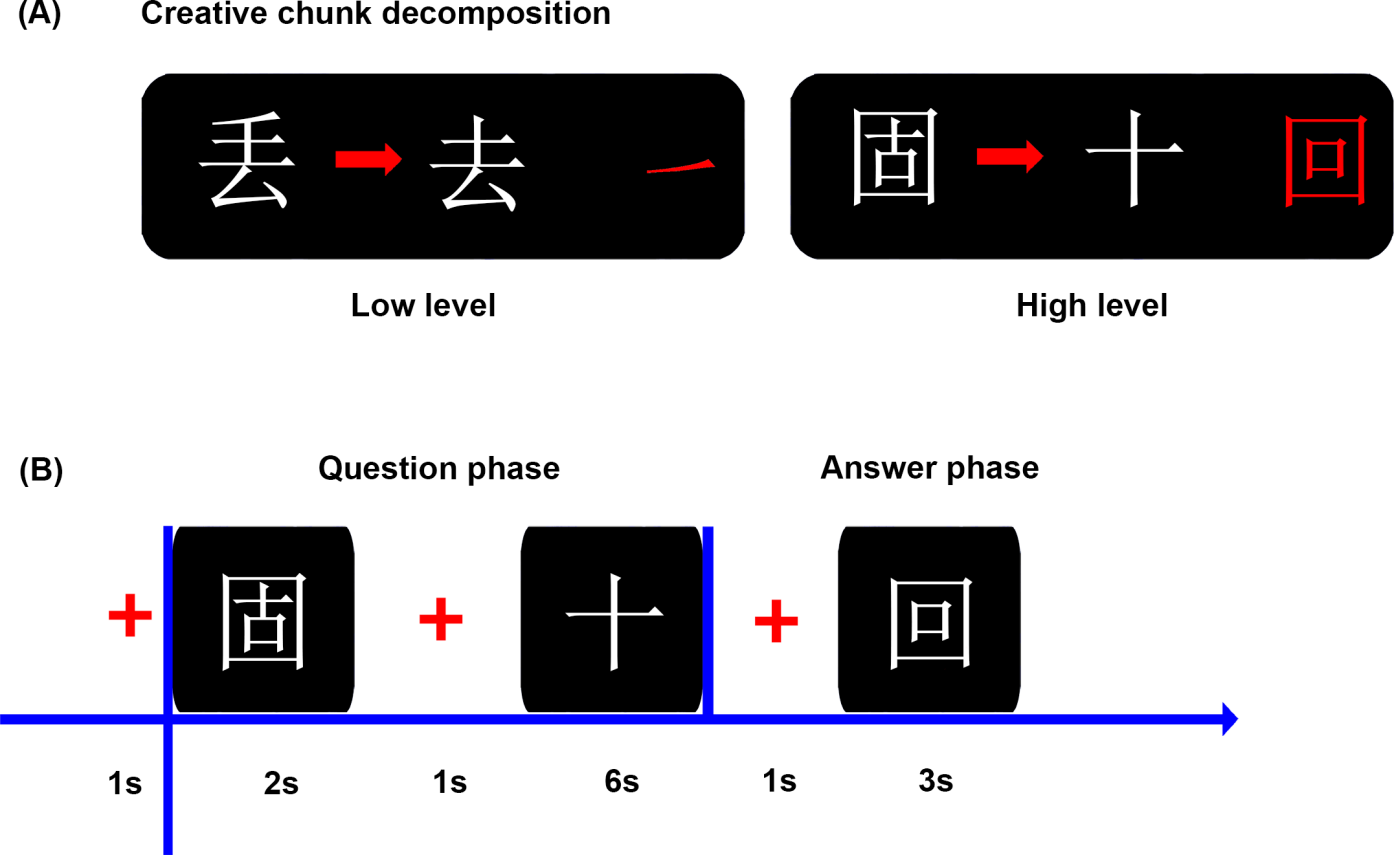
Illustration of the Chinese character chunk decomposition materials and experiment procedure. (A) Examples of two experimental conditions: creative chunk decomposition-low level; creative chunk decomposition-high level. Both conditions were defined by operational definitions (i.e., spatial relationship between the target character and the to-be-removed part or character) and novelty assessment. The to-be-removed part and character are also showed in red color on the right side. (B) The flowchart shows the time course of a trial in behavior experiment. In the question phase, participants were asked to find the to-be-removed portion in their minds, from transforming the given character into the target character as soon as possible. In the answer phase, the answer portion (a part or an intact character) was presented and participants were requested to confirm whether it was the same as the to-be-removed portion found in the question phase or not as soon as possible.

Chinese characters are ideal examples of chunks because of its orthographic structure [35]. In the present study, we designed two types of creative Chinese character chunk decomposition, CCDL and CCDH, based on both operational definition and novelty assessment by a separate sample of 20 participants on a 2-point scale (Fig 1). Specifically, in the CCDL, participants were required to transform a character into a new one by removing a specific part (e.g., a stroke), which was novel to participants but could be solved with a high probability of success. For example, participants were required to decompose the character “丢” (diu, meaning lost) into “去” (qu, meaning to go) by removing the stroke “㇀”. While in the CCDH, participants were asked to transform a character into a new one by removing an intact character (e.g., an isolated character), which was much more novel to participants and could be solved with a low probability of success. For example, participants were required to decompose the character “固” (gu, meaning stable) into “十” (shi, meaning ten) by removing the intact character “回” (hui, meaning to go back). Novelty assessment by paired-samples *t*-test showed that there was significant difference in novelty between the two types of creative Chinese character chunk decomposition [*t* (1,19) = -14.09, *p* < 0.001]. The CCDH was much more novel and difficult than the CCDL. All Chinese characters selected as experimental materials in this study are frequently used characters.

Previous studies have confirmed the validity of using Chinese character chunk decomposition to investigate the creative insight [15, 34]. Creative insight is a sudden comprehension that can lead to a new interpretation of a situation and yield the solution to a problem [1–4]. The Chinese character chunk decomposition task in the present study satisfied the definition of creative insight. For example, we first provided a character, such as “固” (given character), and then we offered the second character, such as “十” (target character). Finally, participants were required to transform the given character into the target character, which constituted the insight problem in the study. Participants were searching the possible solutions in their minds. When they “suddenly” realized the solution to this problem, such as by removing the intact character (“回”) to transform the given character (“固”) into the target character (“十”), they should response as quickly as possible. This “sudden” solution comprehension and chunk decomposing process constituted the main body of insight process to some extent. Besides, the chunk decomposing involved deep representational change which played an important role in creative insight.

### Behavioral task and procedure

We designed two experimental conditions in this study: CCDH and CCDL (Fig 1). Each condition included 80 trials, and the total 160 trials were equally divided into 2 blocks, with a block consisting of 40 trials per condition. The order of the 2 blocks was counterbalanced across participants and trials were randomized in each block. Participants were asked to decompose parts or intact characters from given characters to constitute new characters (target characters). The experiment took place in a sound-attenuated booth. Participants sat in front of the computer screen with their hands located in front of the keyboard. The computer screen resolution was 1024 * 768. The distance between the participant’s head and the screen was about 70 cm.

In each trial, the character need to be decomposed (given character) was presented on the screen for a fixed duration of 2 s and participants were asked to remember this character. After a 1 s fixation cross, another character (target character) was shown on the screen, and participants were required to transform the given character into the target character (insight problem) and find the solution to this problem in 6 s as soon as possible. In other words, participants had 6 s to find the to-be-removed portion and they were instructed to press a button as soon as they constructed the to-be-removed portion in their minds spontaneously, from decomposing the given character into the target character. Then, after a 1 s fixation cross, participants had to decide whether the portion found in their minds previously was the same as the portion (a part or an intact character) that appeared on the subsequent screen within 3 s by pressing a button as soon as possible. Before the experiment, participants performed a training with different stimuli than those used for the formal experiment (Fig 1).

After the behavioral experiment, all participants then underwent an MRI scan during which they were instructed to refrain from head movement and remain awake. The scan was comprised of anatomical imaging (5 min) and resting state imaging (8 min).

### Creative insight assessment

Previous studies have verified that chunk decomposition is a specific form of insightful problem-solving [8, 14, 15, 27–32]. In the present study, we adopted a revised Chinese character chunk decomposition task to assess the degree of creative insight in each participant. Specifically, for each participant, we first calculated the mean RT by averaging the RT of CCDL condition and the RT of CCDH condition. Then we used this mean RT to represent the creative insight score of each participant. Longer (shorter) RT indicated lower (higher) creative insight score. Previous studies have confirmed that it was reasonable to use RT as an index of creative insight [14, 16].

### MRI data acquisition

All images were acquired on a 3T Siemens Trio Tim MRI scanner in South China Normal University. For each participant, the rs-fMRI data, consisted of 240 images (8 mins), was acquired using a gradient-echo-planar imaging (EPI) sequence with the following parameters: repetition time (TR) = 2000 ms, echo time (TE) = 30 ms, thickness = 3.5 mm, field of view (FOV) = 204 × 204 mm^2^, flip angle (FA) = 90°, data matrix = 64 × 64, and 33 axial slices covering the whole brain. In addition, the high-resolution brain structural images were obtained using a T1-weighted 3D magnetization prepared rapid acquisition gradient echo (MP-RAGE) sequence with the following parameters: TR = 1900 ms, TE = 2.52 ms, FA = 9°, data matrix = 256 × 256, FOV = 256 × 256 mm^2^, thickness = 1.0 mm, and 176 sagittal slices covering the whole brain. During the rs-fMRI scan, each participant was requested to relax, close eyes and wake, but not thinking about other things.

### MRI data preprocessing

Data preprocessing and statistical analysis were performed using DPABI (http://rfmri.org/dpabi) [36] based on statistical parametric mapping (SPM8, http://www.fil.ion.ucl.ac.uk/spm/software/spm8/). For each participant, the first ten functional volumes were discarded to allow for scanner equilibration. The remaining images were corrected by slice-timing and realigned for head motion correction. Then, the realigned images were co-registered with the T1-weighted image and normalized to a voxel size of 3 × 3 × 3 mm^3^, using a standard Montreal Neurological Institute (MNI) template. Afterward, the images were smoothed with a 4 mm full width at half maximum (FWHM) Gaussian kernel. At last, we performed signal linear detrending and regressed out the nuisance covariates, including head motion parameters derived from the Friston 24-parameter model, white matter signal, and cerebrospinal fluid signal within each voxel in whole brain. And we have not regressed out the global signal in this study. We estimated 6-parameter head motion and all of subjects satisfied our criteria: translation < 2.5 mm in any plane and angular rotation < 2.5° in any direction during realignment.

### Calculation of ALFF

We calculated the ALFF using the DPABI toolbox with the following steps: (1) For each voxel, the preprocessed time series was transformed to a frequency domain with a fast Fourier transform (FFT) (parameters: taper percent = 0, FFT length = shortest) and the power spectrum was then obtained. (2) The square root of the power spectrum was computed and then averaged across a predefined frequency interval. This averaged square root was termed ALFF at each voxel. The index of ALFF measures the absolute strength or intensity of low frequency oscillations. (3) For standardization purposes, the ALFF of each voxel was divided by the global mean ALFF value to reduce the global effects of variability across the participants [19].

### Statistical analyses

In behavior data analysis, a paired-samples *t*-test was used to assess the differences in novelty between the CCDL condition and CCDH condition, using SPSS software (version 19.0; SPSS, Chicago, IL, USA) and significant level was set at *p* < 0.01 (two-sided). In rs-fMRI data analysis, data analysis was performed with SPM8. In the whole-brain analyses, a multiple linear regression was conducted by using creative insight (defined by mean RT) as the variable of interest to identify regions where ALFF was correlated with individual differences in the level of creative insight after controlling for possible confounding variables, such as age and sex. We determined the surviving clusters at a voxel level threshold of *p* < 0.005 (uncorrected) and cluster level threshold of *p* < 0.05 (FWE corrected) to correct for multiple comparisons.

## Results

### Behavioral data

**Fig 2:**
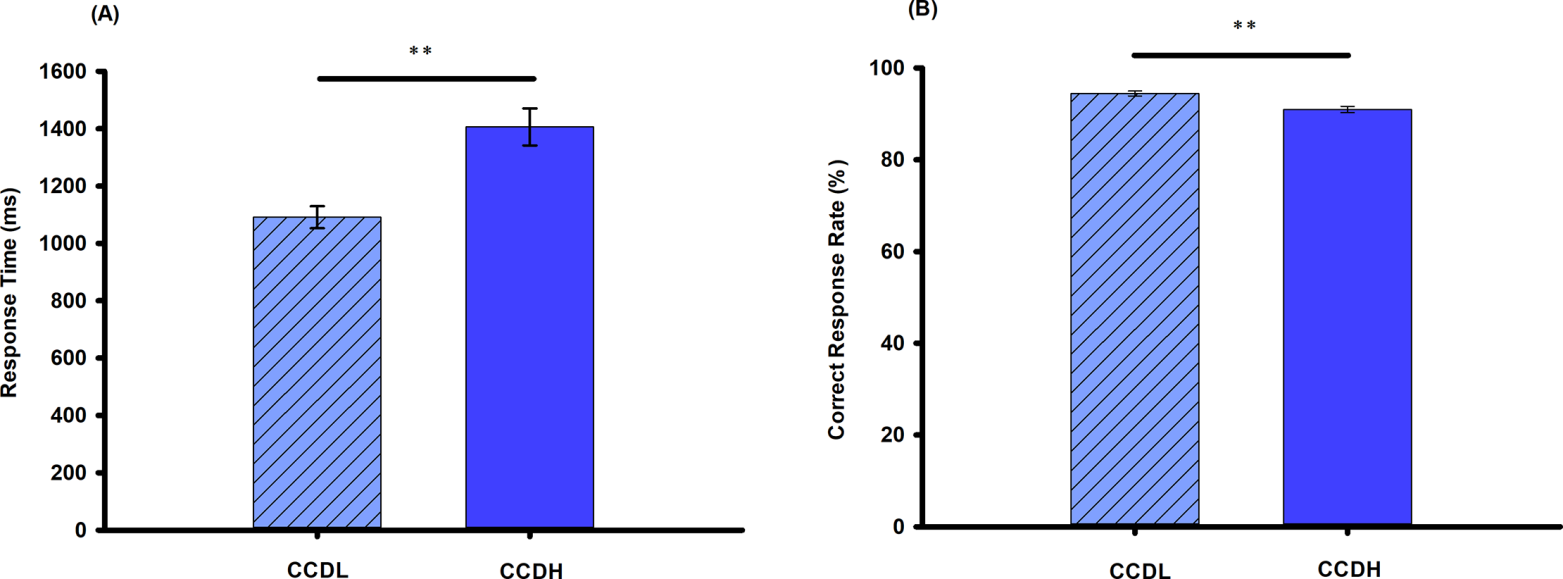
Behavioral results. Panel (A) shows the mean response time and (B) shows the mean correct response rate (%) in the creative chunk decomposition-low level (CCDL) and creative chunk decomposition-high level (CCDH). Error bars correspond to the standard error. The horizontal cap lines with ‘**’ represent *p*-value < 0.01.

Details of the mean RT and correct rate (CR) of each experimental condition were showed on Fig 2. For RT, paired-samples *t*-test revealed that RT of CCDL was significantly longer than that of CCDH [*t* (61) = -9.04, *p* < 0.001]. In addition, CR of CCDL was significantly higher than that of CCDH [*t* (61) = 4.81, *p* < 0.001]. The behavioral data confirmed our predicted hypothesis.

To identify the degree of creative insight in each participant, we used the mean RT of both CCDL and CCDH conditions to represent the creative insight score. The kurtosis and skewness of the creative insight score ranged from −1 to +1, indicating normality of the data [37]. There was no significant correlation between creative insight score and age (*r* = -0.25, *p* = 0.06). And no significant sex difference was observed in the creative insight score [*t* (60) = 0.85, *p* = 0.40].

### Resting-state fMRI data

**Fig 3:**
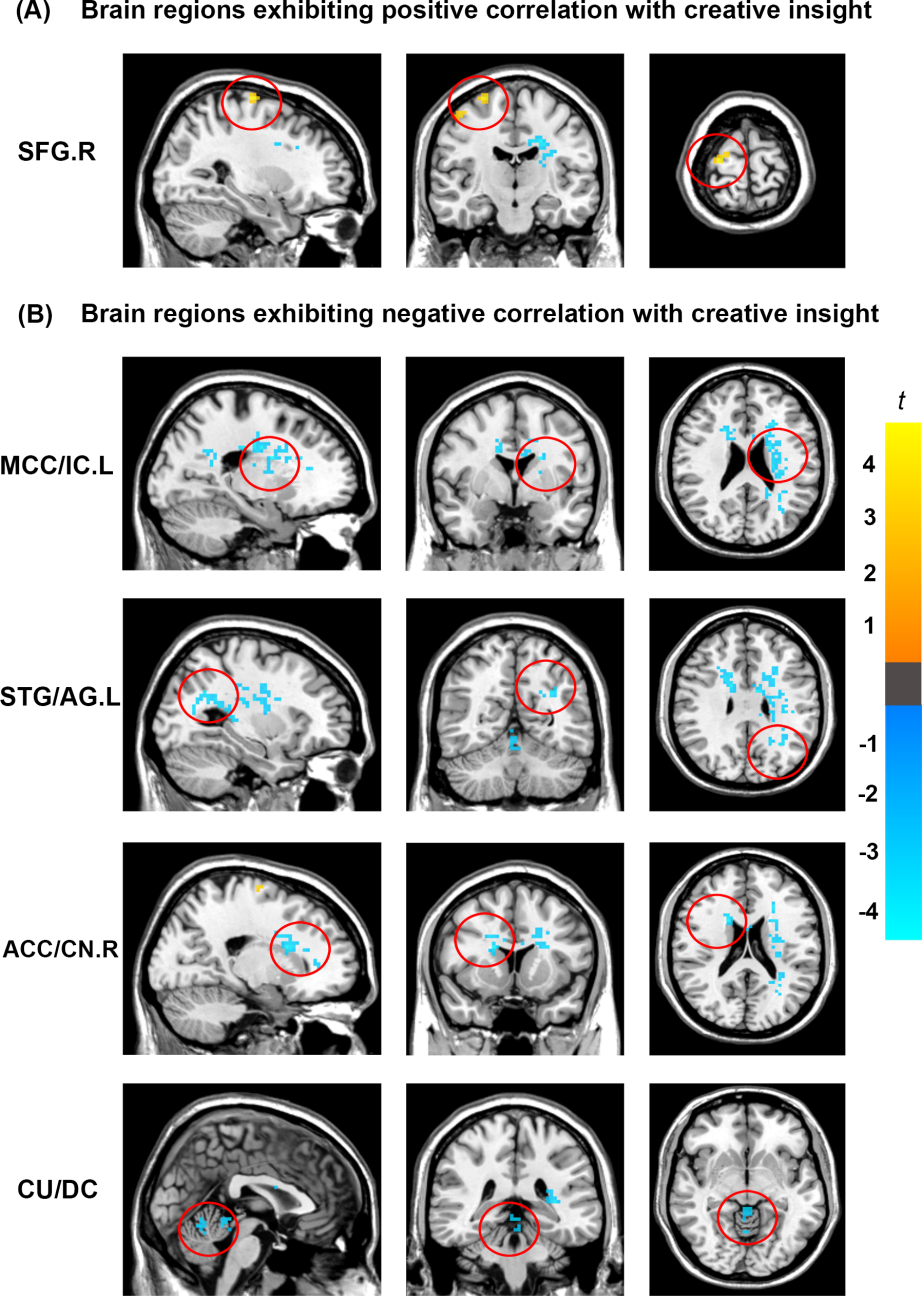
Brain regions showing significant correlation with creative insight. Activation maps are shown at a voxel level threshold of *p* < 0.005 (uncorrected), and cluster level threshold of *p* < 0.05 (FWE corrected). Images are plotted with the MRIcron (https://www.nitrc.org/projects/mricron). (A) ALFF of the right SFG was positively correlated with the creative insight. (B) ALFF of the left MCC/IC, left STG/AG, right ACC/CN, and CU/DC was negatively correlated with the creative insight. Abbreviations: ALFF, amplitude of low-frequency fluctuation; SFG, superior frontal gyrus; MCC, middle cingulate cortex; STG, superior temporal gyrus; ACC, anterior cingulate cortex; CU culmen; IC, insula cortex; AG, angular gyrus; CN, caudate nucleus; DC, declive.

**Fig 4:**
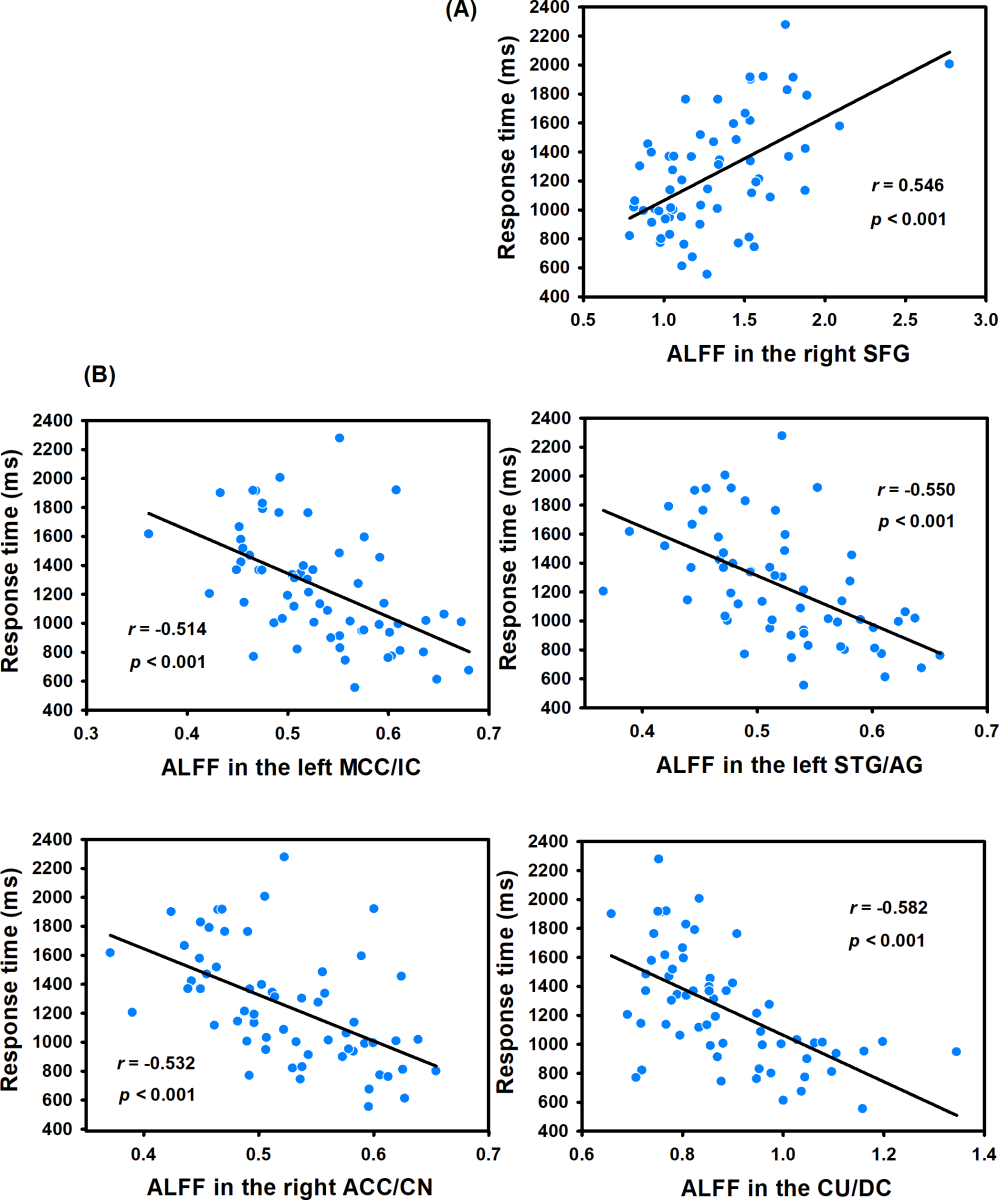
The scatterplot showed correlation between creative insight scores (defined by the mean response time of both creative chunks decomposition) and ALFF. (A) Creative insight scores were positively correlated with ALFF in the right SFG. (B) Creative insight scores were negatively correlated with ALFF in the left MCC/IC, left STG/AG, right ACC/CN, and CU/DC. Abbreviations: ALFF, amplitude of low-frequency fluctuation; SFG, superior frontal gyrus; MCC, middle cingulate cortex; STG, superior temporal gyrus; ACC, anterior cingulate cortex; CU culmen; IC, insula cortex; AG, angular gyrus; CN, caudate nucleus; DC, declive.

In this study, we correlated the creative insight scores with the ALFF value of each voxel across the whole brain to explore the neural correlates of creative insight. After controlling for age and sex, the creative insight scores were significantly and positively associated with one cluster located in the right SFG. Moreover, the creative insight scores were significantly and negatively associated with four clusters located in the left middle cingulate cortex/insula cortex (MCC/IC), left STG/angular gyrus (AG), right ACC/caudate nucleus (CN), and culmen/declive (CU/DC). Details of these clusters were showed on Table 1 and Figs 3 and 4.

**Table 1:** Brain regions showing significant correlation with creative insight. Clusters were obtained at a voxel level threshold of *p* < 0.005 (uncorrected) and cluster level threshold of *p* < 0.05 (FWE corrected) to correct for multiple comparisons.

Regions	Side	Cluster size	MNI coordinates			*t* value
		(voxels)	x	y	z	
Positive correlation						
SFG	RH	55	27	-15	72	5.00
Negative correlation						
MCC/IC	LH	336	-21	0	24	-4.88
STG/AG	LH	98	-30	-57	30	-4.00
ACC/CN	RH	89	18	12	21	-4.73
CU/DC	L, RH	66	0	-36	-6	-3.79

Coordinates are the stereotactic space of the Montreal Neurological Institute. The *t* value corresponds to the peak voxel showing greatest statistical difference within a cluster. Abbreviations: SFG, superior frontal gyrus; MCC, middle cingulate cortex; STG, superior temporal gyrus; ACC, anterior cingulate cortex; CU culmen; IC, insula cortex; AG, angular gyrus; CN, caudate nucleus; DC, declive; LH, left hemisphere; RH, right hemisphere.

## Discussion

In this rs-fMRI research, we explored the potential contribution of spontaneous brain activity to creative insight. The analyses showed that creative insight scores were positively correlated with ALFF in the SFG, while negatively correlated with ALFF in the MCC/IC, STG/AG, ACC/CN, and CU/DC, indicating that these brain regions were associated with creative insight.

These findings showed that creative insight scores were significantly positively correlated with ALFF in the SFG. Previous studies demonstrated the importance of SFG in executive functions, especially in working memory, mental manipulation, and spatially oriented processing [38, 39]. Furthermore, another previous study indicated that the SFG supported planning and motivation [40]. In addition, previous studies have suggested that the creative insight was linked to mental manipulation, task-related associations [10, 34]. For example, previous studies showed that representation change, and forming task-related associations were the crucial process of creative insight [10, 34]. Therefore, in the present study, the result that ALFF in the SFG predicted creative insight significantly may indicate a stable relationship between creative insight and representation change and forming task-related associations. Moreover, in a task-fMRI study, Huang et al. [14] found that the SFG was activated in insight problem-solving, which was partially consistent with our current study.

We also detected that creative insight was negatively linked with ALFF in the cingulate cortex, including the ACC and MCC. The ACC extends across CN and the MCC extends across IC in the present study. The ACC emerged as a hub region of conflict detection, restructuring processes, performance monitoring, and affective functions [14, 41, 42]. Similarly, the MCC has been suggested to be involved in response selection, monitoring and affective processing [43, 44]. Furthermore, previous studies have indicated that the creative insight was related to inhibiting the predominant irrelevant mental representations, establishing the new representation in a goal-directed way, and positive emotional experience [11, 45]. For instance, Bowden and Jung-Beeman [46] found that problem solvers experienced their solutions of insightful problems as sudden and surprising, which called the “*Aha*! moment”. Subramaniam et al. [11] emphasized that problem solvers were required to detect competing solution candidates, rely less on dominant associations, and break and establish set to solve the insight problem. Thus, the finding that ALFF in the ACC and MCC predicted creative insight may suggest a relationship between creative insight and conflict monitoring, breaking and establish mental sets, and emotional experience. In addition, we also found creative insight was negatively correlated with ALFF in the CN and IC. The CN was the core region of the procedural memory system [46, 47], and could be related to the habit formation [48], novelty assessment [15], and sequence learning [49]. The role of the IC could be related to emotional experience, and processing uncertainty in the context of decision making [50]. Previous studies indicated that problem solvers come to an impasse, when trying to solve an insight problem, perhaps because they were misled by ambiguous in formation in the problem [45, 51]. Moreover, creative insight included processes to break mental sets and to form new task-related associations [52]. Thus, the activations in IC and CN could be related to emotional experience, novelty assessment, and forming novel associations in the insight problem-solving. Our findings were consisted with previous task-related fMRI studies of creative insight [11, 13–15]. In a word, ALFF of the resting-state brain activity could predict creative insight.

In addition, we also found that creative insight was negatively correlated with ALFF in the STG and the CU. In the present study, the STG extends across AG, and the CU includes parts of the DC. The STG played a critical role in representing spatial awareness, and object-related exploration [53, 54]. The AG was an association area related to manipulate mental representations, reasoning and comprehension [55, 56]. The STG and AG activations indicated that insight problem solving required more efforts, including information comparison, solution exploration and selection, and reasoning, which was in line with previous studies of insight problem-solving [13, 45]. Therefore, the result that ALFF in the STG and AG predicted creative insight significantly may indicate a relationship between creative insight and object-related exploration and reasoning. The regions of CU and DC, which belong to cerebellum, have been previously reported to be related to language processing [57, 58]. Previous studies demonstrated that participants with a high creative performance showed significantly greater neural activity in the cerebellum, including the CU and DC, which was consisted with our findings in the present research [59, 60]. In this study, we used the Chinese character chunk decomposition as the experiment materials, which would probably recruit the language processing, then activate the CU and DC. However, it is noteworthy that the explanation of the above activated brain regions was based on findings from previous studies. Further research is needed to explore this possibility in greater detail.

Finally, with the task of creative chunk decomposition in this rs-fMRI study, we sketched a preliminary model of creative insight which defines the functional role of each region. We speculated that the activations in the STG and AG involved in exploring, reasoning, and looking for the insightful solution which is meaningful and target-related at the same time. The ACC, MCC and SFG were related to inhibiting the predominant irrelevant task representations, restructuring the representations in a goal-directed way, and established the novel representations. Furthermore, the activations in the ACC, MCC and IC could also be partly related to the “Aha!” experience accompanied with insight problem solving. CN played an important role in the evaluation of novel and insightful solutions and forming novel associations in creative insight.

There was a limitation in the present study. For each participant, we defined the degree of creative insight by the mean RT of both creative chunk decompositions, which was controversial. This approach was reasonable to some extent since lower creative insight score of the participant indicated more difficulty and longer RT in the chunk decomposition. And previous studies of chunk decomposition have demonstrated the validity of RT as an index partly reflecting the extent of creative insight [14, 15, 33]. However, the RT might simply reflect the task difficulty, not insight itself. Thus, in the study, we also used the mean CR to characterize the degree of creative insight. Higher (lower) CR indicated higher (lower) creative insight score. However, results of the whole brain analysis showed that only one cluster of middle occipital gyrus was significantly correlated with the creative insight. The reason for this might be that all the participants had a relative high CR in the study. Using the CR as the index might be possibly hard to represent the individual variations in the degree of creative insight. It seems that RT might be a more sensitive index of creative insight than CR. In the future, a more accurate index or task is needed to characterize the creative insight.

In conclusion, using the rs-fMRI and chunk decomposition paradigm, we explored the neural correlates of creative insight. The findings demonstrate that ALFF in the SFG, MCC/IC, STG/AG, ACC/CN and CU/DC can be used to predict individual differences in creative insight. Furthermore, the ACC, MCC and IC are implicated in emotional experience in creative insight. The ACC, MCC and SFG involved in conflict monitoring, breaking mental sets, and forming task-related associations. The STG and AG were responsible for exploring, reasoning, and looking for insightful solutions, with an evaluation of novel solutions by the CN. In a word, the present study is conductive to further understand the cognitive processing and neural correlates of creative insight.

## Supporting information

S1 File Illustration of the experiment behavior data and fMRI data. (A) Behavior data showed the gender and age of each participant and the RT, CR in the CCDL and CCDH condition of each participant. (B) fMRI data showed the mean ALFF brain map of each participant. Abbreviations: RT, response time; CR, correct rate; CCDL, creative chunk decomposition-low level; CCDH, creative chunk decomposition-high level; ALFF, amplitude of low-frequency fluctuation.
(ZIP)

## Abbreviations

fMRI: functional magnetic resonance imaging
rs-fMRI: resting-state fMRI
ALFF: amplitude of low-frequency fluctuation
SFG: superior frontal gyrus
MCC: middle cingulate cortex
IC: insula cortex
STG: superior temporal gyrus
AG: angular gyrus
ACC: anterior cingulate cortex
CN: caudate nucleus
CU: culmen
DC: declive
IFG: inferior frontal gyrus
MFG: middle frontal gyrus
BOLD: blood oxygenation level-dependent
CCDL: creative chunk decomposition-low level
CCDH: creative chunk decomposition-high level
RT: response time
MNI: Montreal Neurological Institute
FWHM: full width at half maximum
FFT: fast Fourier transform
CR: correct rate
