# Correction: Neural correlates of creative insight: Amplitude of low-frequency fluctuation of resting-state brain activity predicts creative insight

**DOI:** 10.1371/journal.pone.0213212

**Published:** 2019-02-27

**Authors:** Jiabao Lin, Xuan Cui, Xiaoying Dai, Yajue Chen, Lei Mo

The authors reused Fig 1A and 2 from a previously published related article in *Frontiers in Human Neuroscience* [1] prior to the publication of their *PLOS ONE* article [2], but did not cite or discuss this related work. In addition, Fig 2 in both articles appear similar. However, the underlying data are different for each Fig 2. Specifically, data used in both figure came from the same dataset but with different sample sizes. [2] reported results from all 62 participants. Meanwhile, [1] reported results from 50 participants. The authors apologise for this oversight.

The authors wish to clarify the relationship between the two articles: The authors tested the relationship between low frequency oscillations and creative insight; measured ALFF and reported the results in the *PLOS ONE* article [2]. However, the authors also tested the relationship between local connectivity and creative insight; measured ReHo and reported results in the *Frontiers in Human Neuroscience* article [1].

The study published in [1] was based on a subsample (80%) of the population studied in [2] and the creative insight tasks in both studies were identical. For each ROI, the authors estimated the correlations between ALFF and ReHo values. Results showed that ALFF values are significantly correlated with the ReHo values in the CU/DC (r = 0.364, p = 0.004), MCC/IC (r = 0.549, p < 0.001), STG/AG (r = 0.608, p < 0.001), ACC/CN (r = 0.503, p < 0.001), and SFG (r = 0.750, p < 0.001). Thus, the results of both studies were highly correlated, and the data have been provided below as a S1 File.

The authors scanned 62 participants and all of them passed the screen tests (e.g., head motion) and ALFF results from these participants has been published in [2]. However, in [1], the authors reported ReHo results of 50 participants out of the total 62 participants. 12 participants were excluded as they did not pass additional screen tests required for ReHo.

The results of [2] show that ALFF in the SFG positively predicted creative insight, while ALFF in the MCC/IC, STG/AG, ACC/CN, and CU/DC negatively predicted creative insight. The results of [1] showed that ReHo in the ACC/CN and STG/AG/IPL negatively predicted creative insight.

The activated brain regions in both studies overlapped in the clusters of ACC/CN and STG/AG as both are hub regions for creative insight. In addition, using the ALFF method in [2], creative insight induced additional brain activations in the SFG, MCC/IC, and CU/DC compared to [1].

Since ALFF and ReHo reveal different neural bases of creative insight, the authors reported them separately in two independent papers.

## Supporting information

S1 File Illustration of the ALFF values, the ReHo values, and the Pearson correlation between the ALFF and ReHo in each ROI. Results showed that there was a significant and positive correlation between the ALFF and ReHo in each ROI. Notably, the ROIs were derived from the results of the PLOS ONE study.
(XLSX)

## References

[1] Lin J, Cui X, Dai X and Mo L (2018) Regional Homogeneity Predicts Creative Insight: A Resting-State fMRI Study. Front. Hum. Neurosci. 12:210 10.3389/fnhum.2018.00210 29875645 PMC5974035

[2] Lin J, Cui X, Dai X, Chen Y, Mo L (2018) Neural correlates of creative insight: Amplitude of low-frequency fluctuation of resting-state brain activity predicts creative insight. PLoS ONE 13(8): e0203071 10.1371/journal.pone.0203071 30161187 PMC6117043

